# Curvilinear Kirigami Skins Let Soft Bending Actuators Slither Faster

**DOI:** 10.3389/frobt.2022.872007

**Published:** 2022-05-03

**Authors:** Callie Branyan, Ahmad Rafsanjani, Katia Bertoldi, Ross L. Hatton, Yiğit Mengüç

**Affiliations:** ^1^ Collaborative Robotics and Intelligent Systems Institute, Oregon State University, Corvallis, OR, United States; ^2^ Sandia National Laboratories, Albuquerque, NM, United States; ^3^ Center for Soft Robotics, SDU Biorobotics, University of Southern Denmark, Odense, Denmark; ^4^ John A. Paulson School of Engineering and Applied Sciences, Harvard University, Cambridge, MA, United States; ^5^ Meta Reality Laboratory–Research, Redmond, WA, United States

**Keywords:** soft robotics, kirigami, bioinspiration, biorobotics, mechanical design, mechanical metamaterials, locomotion

## Abstract

The locomotion of soft snake robots is dependent on frictional interactions with the environment. Frictional anisotropy is a morphological characteristic of snakeskin that allows snakes to engage selectively with surfaces and generate propulsive forces. The prototypical slithering gait of most snakes is lateral undulation, which requires a significant lateral resistance that is lacking in artificial skins of existing soft snake robots. We designed a set of kirigami lattices with curvilinearly-arranged cuts to take advantage of in-plane rotations of the 3D structures when wrapped around a soft bending actuator. By changing the initial orientation of the scales, the kirigami skin produces high lateral friction upon engagement with surface asperities, with lateral to cranial anisotropic friction ratios above 4. The proposed design increased the overall velocity of the soft snake robot more than fivefold compared to robots without skin.

## 1 Introduction

Soft robots and limbless animals must break symmetry to achieve locomotion—through changing the shapes of their bodies and through the interaction of their skin with environments. Soft robots are particularly well suited for massive shape changes, but these changes result in large material deformations that make the attachment of skins challenging. Looking for existence proofs in nature to address this gap, we find snakes combine a malleable body coupled with specialized skins. Among snakes, lateral undulation is a typical gait strategy to leverage the combined benefits of their body and skin. As a result, snakes exist in nearly all ecological niches, from aquatic, terrestrial, to arboreal. To achieve undulatory locomotion, snakes propagate a body wave from head to tail (Gans, 1962) and their scales engage with asperities in the environmental surfaces resulting in necessary frictional anisotropy (Hu et al., 2009). Thus, any synthetic skin applied to snake-like robots must replicate the same ratio of lateral to longitudinal friction while permitting the body to slither.

Snakes primarily rely on undulatory locomotion to navigate through complex terrains. Rigid snake robots performing lateral undulation have been widely developed, but since they often use wheels to generate lateral forces (Hirose, 1993; Crespi and Jan Ijspeert, 2006), they lack the compliance of biological snakes. Soft snake robots performing lateral undulation have been developed to increase the deformability of the robot through the incorporation of soft materials (Onal and Rus, 2013; Branyan et al., 2017). There is biological evidence that lateral reaction forces are necessary for generating propulsive forces during undulatory locomotion (Hu et al., 2009) with measured lateral to longitudinal friction anisotropy values that can reach up to about 2:1 (Gray and Lissmann, 1950; Berthe et al., 2009; Hu et al., 2009; Benz et al., 2012; Marvi and Hu, 2012; Abdel-Aal, 2013).

Current snake-inspired robots fail to accurately mimic the locomotion of biological snakes because they neglect the role of frictional interactions in the robot skin design. To enhance the performance of soft slithering robots, we should carefully engineer the friction behavior of the skin of soft robots and synchronize it with the body deformation.

In recent years, inspired by the Japanese art of paper cutting, engineers adopted kirigami to create flexible metasurfaces by perforating an array of cuts into thin elastic sheets to achieve a wide range of mechanically-programmable physical properties (Blees et al., 2015; Lamoureux et al., 2015; Shyu et al., 2015; Zhang et al., 2015; Isobe and Okumura, 2016; Rafsanjani et al., 2017; An et al., 2020; Babaee et al., 2020, 2021; Hong et al., 2022). Stretching kirigami skins triggers elastic instabilities in thin ligaments and transforms the smooth surface of the skin into a 3D texture that has the potential to imbue soft robots with new functionalities (Rafsanjani et al., 2019a). Kirigami lattices on thin elastic sheets are ideal for integrating with elastomeric actuators because they can be designed to deform with the soft actuator. The standard kirigami lattice that allows for stretching of the thin sheet and combined with an extending soft actuator has enabled the soft robots to achieve crawling (Rafsanjani et al., 2018; 2019b), and burrowing (Liu et al., 2019). Altering the kirigami lattice to account for bending as well as elongation and combined with bending soft actuators has enabled soft robots to achieve undulating locomotion (Branyan et al., 2020). Kirigami metasurfaces have also been used as soft grippers (Yang et al., 2021).

The work presented here improves the locomotion of a soft snake robot by implementing an enhanced kirigami pattern that increases the lateral-cranial friction ratio on a snake-inspired skin. Based on results from previous work on a snake-inspired kirigami skinned soft robot (Branyan et al., 2020), we know that increasing frictional anisotropy of the soft snake robot increases the velocity of locomotion. A soft actuator without skin relies on adhesion between the elastomeric material and the surface, rather than sliding friction resulting in isotropic reaction forces. We look to increase the velocity of lateral undulation in this work by controlling the direction of reaction forces from the scales as the body deforms through the design of the lattice of arrayed cuts.

The proposed kirigami design follows a curved pattern, that when wrapped around a soft, fiber-reinforced, bending actuator uses enhanced directional friction to promote slithering of the robot. Bending is achieved by creating a gradient of hinge widths in the lattice pattern, which reduces the overall axial stiffness of the skin when wrapped around an actuator. As the scales rotate laterally (pointing out from the body to the sides) during bending, the anisotropic ratio of friction in the caudal-direction (towards the tail, against the scales) to friction in cranial-direction (towards the head, with the scales) of the system will decrease as fewer scales are oriented along the spine. This trade-off is justified because of biological evidence that lateral reaction forces are necessary for the production of lateral undulation (Hu et al., 2009; Zhang et al., 2021). Biological measurements show a lateral-cranial frictional anisotropy ranging from 1 to 1.72 (Gray and Lissmann, 1950; Berthe et al., 2009; Hu et al., 2009; Benz et al., 2012; Marvi and Hu, 2012; Abdel-Aal, 2013). Thus, we hypothesized that scales approaching the fully lateral orientation would maximize locomotion efficacy of slithering, even at the cost of reduced caudal-cranial frictional anisotropy. To test this prediction, we introduced curved lattices designed to orient the scales laterally to maximize the lateral reaction force, at the cost of decreased longitudinal reaction forces. The resulting locomotion experiments corroborate our hypothesis—the velocity of slithering soft robots is highest with skins that maximize lateral-friction.

## 2 Design

At their simplest, soft pneumatic actuators are composed of a hyperelastic membrane filled with pressurized fluid. The addition of selectively patterned material directs the isotropic expansion of pressurized gas to do useful anisotropic work. The soft snake robot combines four of these simple fiber-reinforced actuators to form two bidirectional bending actuators that replicate undulatory kinematics. In contrast to its anisotropic kinematics, the isotropic elastomeric skin material (EcoFlex^∗^00–30) does not produce any directional friction forces when in contact with the ground. Any locomotion produced relies on the out-of-phase activation of the two bidirectional bending actuators, and the adhesion between the elastomer and the terrain. Achieving both high absolute lateral friction forces and high caudal-cranial frictional anisotropy through the implementation of a snake-inspired skin greatly increases the velocity of the robot when using lateral undulation. Without a snake-inspired skin, the robot is slow to move across solid surfaces during lateral undulation, and has the tendency to drift sideways.

**FIGURE 1:**
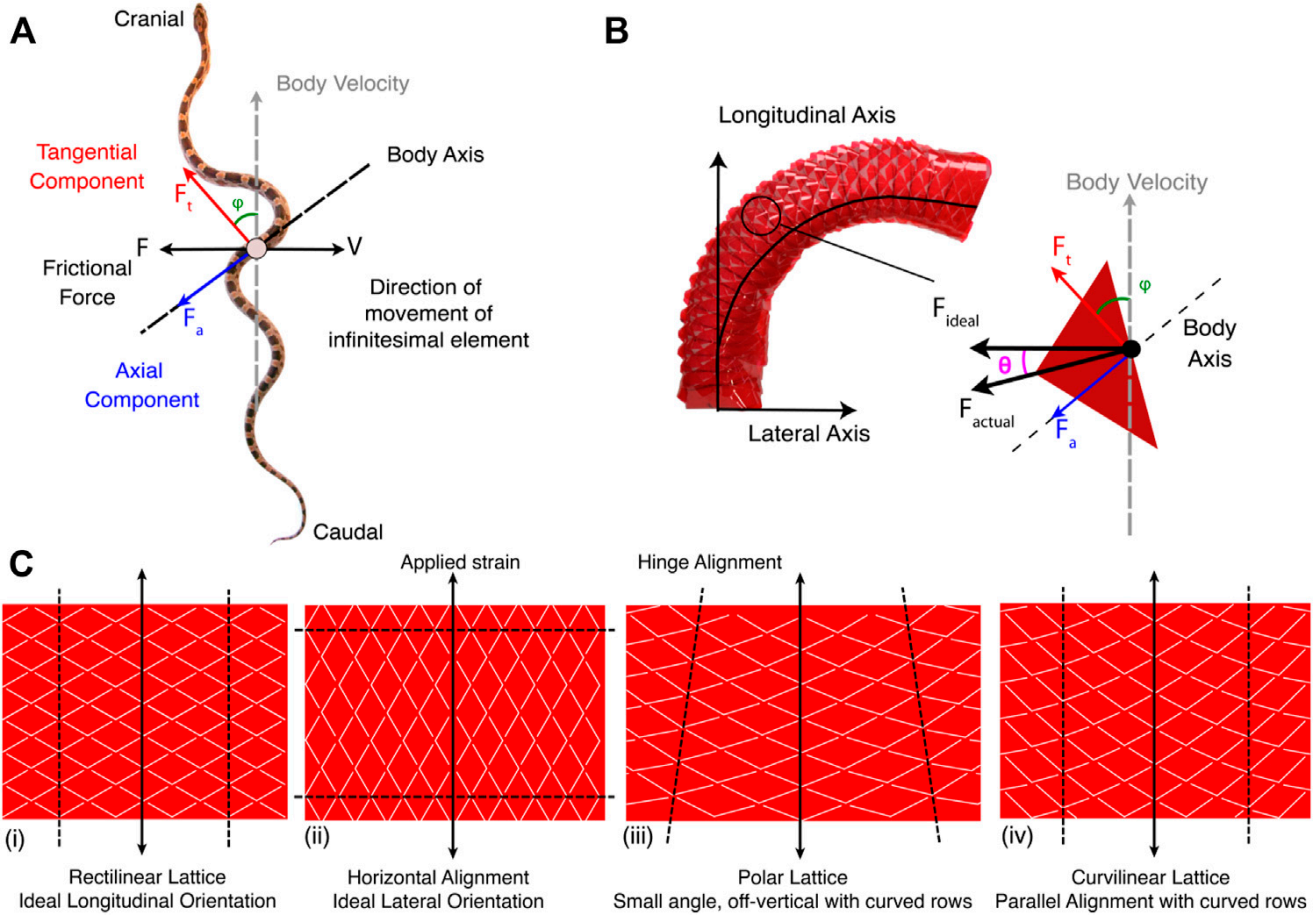
**(A)** Free body diagram of an infinitesimal element of a snake body during lateral undulation (Calisti et al., 2017). (Corn snake pictured (Backwater Reptiles Blog, 2020)). **(B)** Free body diagram of a scale on a bending actuator. **(C)** Lattice patterns for various scale orientation strategies: (i) is a standard kirigami pattern that stretches longitudinally but does not bend, and was used to let soft actuators crawl (Rafsanjani et al., 2018), (ii) the same standard pattern ideally will not stretch in the lateral orientation, (iii) the polar lattice proposed in this paper allows for bending but the scale sizes change along the length of the body, and (iv) the curvilinear lattice proposed in this paper allows for bending and has longitudinally-invariant scales, but is limited to smaller angles of in-plane rotation.

Lateral undulation is characterized by a traveling sinusoidal wave where an infinitesimal mass element along the sinusoid body is moving in a lateral direction while the overall body velocity is forwards. A free body diagram of an infinitesimal mass element is shown in Figure 1A). The friction force felt by the mass element is in the opposite direction of the element’s velocity and includes axial and tangential components with respect to the body axis of the snake at that mass element. Forward propagation of the snake is only possible when *F*_*t*_ cos *φ* > *F*_*a*_ sin *φ* (Calisti et al., 2017). The micro-mechanics of the interaction between the skin and the terrain is outside the scope of this work, so we assume that the friction force produced by an infinitesimal mass element is in the direction of a single scale tip on the skin. Similar assumptions have been made previously to model and measure contact forces to determine if snakes move their scales to increase friction (Marvi et al., 2016). A free body diagram of the forces acting on a scale is shown in Figure 1B). The actual friction force, *F*_actual_ is along the scale tip and is separated by an angle *θ* from the ideal friction force vector, *F*_ideal_ as established in Figure 1A). The new kirigami lattices presented in this work were designed to minimize *θ* so that as

$$\theta \to 0,\;F_{\text{actual}}\to F_{\text{ideal}}$$ (1)

Typical kirigami lattices are arranged in a rectilinear grid (Figure 1Ci). A rectilinear pattern ensures that the scale tip is always aligned with the body axis of the actuator during locomotion, meaning *θ* = *ϕ*. Rectilinear lattices still produce useful friction forces that improve the velocity of the robot enacting lateral undulation (Branyan et al., 2020), but there is no tangential component of the friction to optimize interaction during lateral undulation. Since we want to minimize *θ*, we need a strategy to control the orientation of the scale tips on the skin. The ideal lattice pattern would take the rectilinear pattern and rotate it by 90° as shown in Figure 1Cii so the scale tips are pointed laterally to start. However, out-of-plane buckling can only occur if the hinges are mostly aligned with the direction of applied strain. If the scales are oriented 90° from the direction of the applied strain, the hinges cannot buckle, and the scales do not pop out. By patterning the scale tips along a curve rather than a straight line the scale tips can have an initial rotated orientation, that when activated, approaches the ideal friction vector.

Two lattice structures were developed to alter scale orientation: a polar lattice (Figure 1Ciii) and a curvilinear lattice (Figure 1Civ). The polar lattice is defined by polar coordinates where hinges are located based on an (*R*, *θ*_*lattice*_) pair. The curvilinear lattice has hinges located along a radius spaced by parallel columns as they are for the rectilinear lattice. A third skin was developed using the curvilinear lattice with an additional circumferential translation of the scale tip to push the initial orientation further towards the lateral axis (see Supplementary Figure S1B). The angle between the ideal friction force vector and the actual vector, *θ* can then be characterized as a function of the bending angle of the actuator (*γ*) and the initial scale tip rotation (*θ*_0_) which is a function of the radius of the row (*R*) and the local circumferential translation (*ζ*):

$$\theta =f\left(\text{γ},\;\theta_{0}\left(R,\zeta \right)\right)$$ (2)

A full geometric definition of the kirigami skins can be found in the Supplementary Material.

In addition to redefining kirigami skins to allow for ideal scale orientation, we also needed to ensure the new skins allowed for bending of the actuators upon inflation. A basic rectilinear lattice with a triangular scale profile does not allow for bending greater than 10° unless a strategy for decreasing longitudinal stiffness is employed. One simplistic strategy is to make the hinge width as small as possible and the cut profile as large as possible. However, practical material strength constraints limit the implementation of this strategy: if the hinge width is too small, the lattice will easily rip upon contact with a surface. Since the skin is implemented on a robot for practical environmental interactions, the scales need to have a relatively high geometric stiffness when engaged with surface asperities to provide adequate reaction forces to push off against, which decreases with decreasing hinge width. A gradient of hinge widths can be implemented on the rectilinear lattice that has large hinge widths along the bottom of the actuator to ensure stiff scales engage with asperities, but decreases circumferentially to reduce the overall axial stiffness to achieve bending. The gradient is defined by decreasing the hinge width in the ventro-lateral and dorsal sections of the skin. The curved lattices have an inherent gradient of hinge sizes as well as changes in scale size and shape ensuring bending capabilities without any additional design modifications. We have designed curved lattices to ensure the orientation of scales approach the ideal configuration for maximizing lateral pushing.

## 3 Results and Discussion

### 3.1 Deformation

**FIGURE 2:**
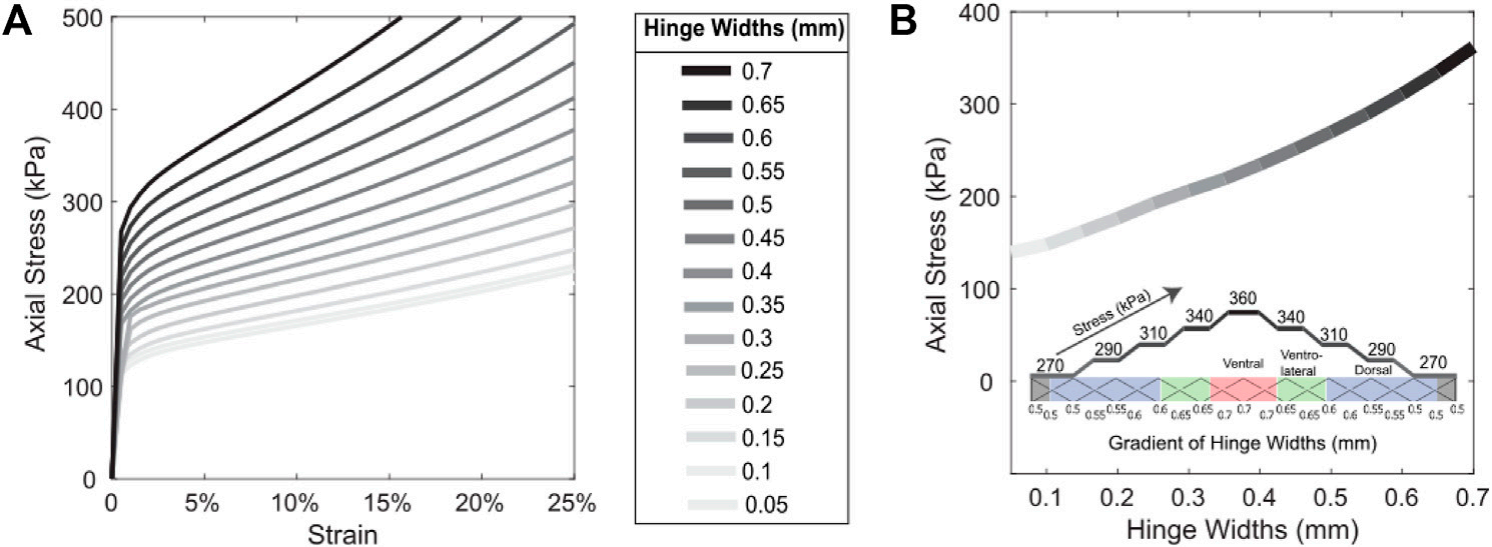
**(A)** Stress-strain curves for rectilinear lattice and triangular cut profiles at different hinge widths. **(B)** Axial stress across hinge widths used to select hinge widths. The inset shows the hinge width gradient across rectilinear lattice introduced to produce bending.

**FIGURE 3:**
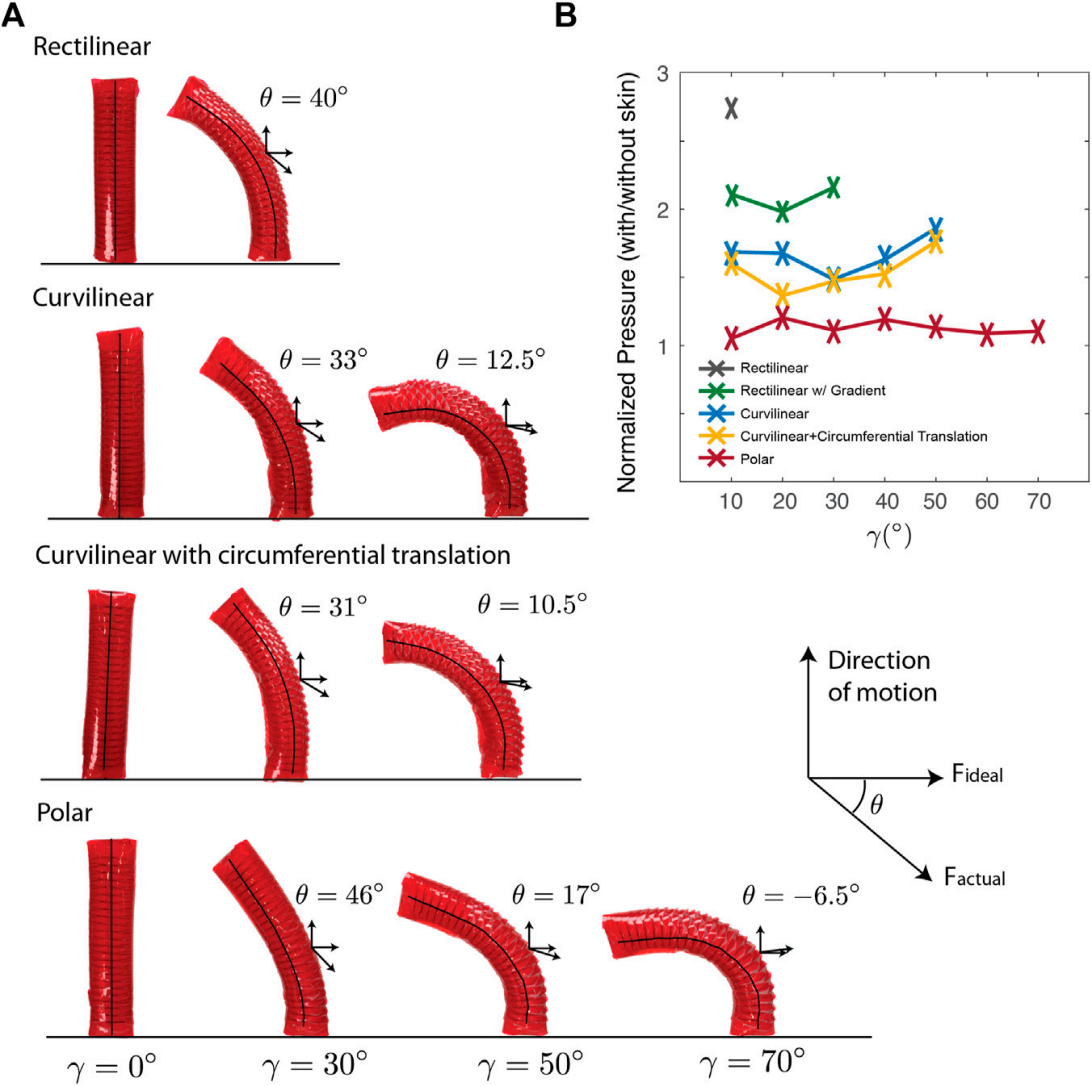
**(A)** In-plane scale rotation for each skin under bending: (i) rectilinear achieves bending through the non-ideal behavior of the kirigami material that can strain and twist out-of-plane, (ii) curvilinear lattice with inherent hinge gradient, (iii) curvilinear lattice with additional circumferential translation of scale tip further decrease *θ*, (iv) polar lattice where scales actually over-rotate at the largest bend angle. **(B)** Normalized pressure-bend angle relationship.

The stress-strain characterization, determined using finite element analyses, of a rectilinear lattice with triangular scale profiles at different hinge widths is shown in Figure 2A. Figure 2B shows the stress across hinge widths when strained at 5% which is about half the maximum strain achieved on the skins. These analyses were used to select the hinge widths used in the gradient on the rectilinear lattice shown in the inset of Figure 2B. Finally, Figure 3B shows the normalized pressure-bend angle relationship of the four skins employed. Despite the gradient of hinge widths allowing for bending, the curved lattices, which have a more dramatic gradient of hinge widths and varying scale shape and size, allowed for larger bend angles. The maximum bend angle achieved by the rectilinear lattice was 30°. The curvilinear lattice with and without the additional circumferential translation had a maximum bend angle of 50°, while the polar lattice had a maximum bend angle of 70°. The polar lattice had the most bending due to the large scale size, as well as an axial stiffness gradient caused by increasing the scale size for each additional row of scales. Figure 3A shows the difference in angles of the lateral (ideal) vector for each skin type on a scale located in the ventro-lateral section of the skin. The smaller the angle shown, the more ideal the interaction with the asperity should be for lateral pushing. The curvilinear lattice has the smallest angle, which means it should produce the highest lateral friction. The additional circumferential translation of the scales on the curvilinear lattice resulted in a 2° reduction in the angle of scale rotation which should further increase lateral friction.

### 3.2 Friction

**FIGURE 4:**
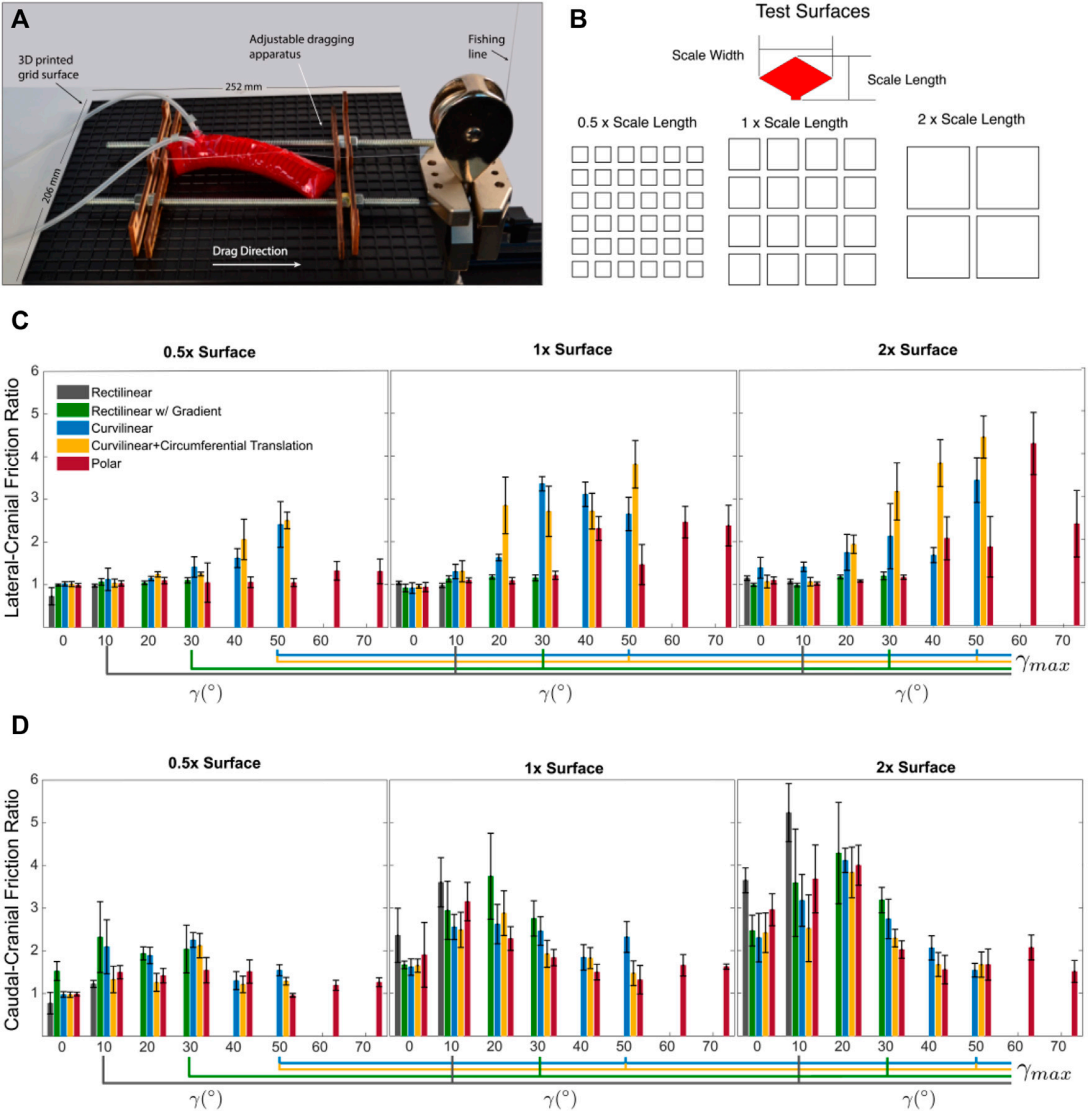
**(A)** Experimental apparatus for measuring directional friction. Actuator with polar lattice skin on 2x scale length surface. **(B)** Test surface grid spacing normalized by scale length. **(C)** Lateral-cranial friction ratio. **(D)** caudal-cranial friction ratio.

Directional friction tests (cranial, caudal, and lateral) were performed using a dragging apparatus (see Figure 4A) attached to a 10N load cell on a universal testing machine (Mark-10 Corp, NY) with a monofilament nylon cord (0.25 mm, Beadalon^®^). Friction force was measured by recording the reaction force of the nylon cord against the displacement of the soft actuator wrapped in a kirigami skin as it was dragged across a rough surface. For each skin type, the actuator was inflated and held at a specified pressure corresponding to the desired bend angle. The dragging apparatus was fit to the shape of the actuator by adjusting the position of the lead screws and two planes placed at two extremes of the actuator. The apparatus was positioned such that it did not drag along the test surface and did not contribute to the drag force measured on the Mark-10. Figure 4A shows the actuator in the cranial orientation. The actuator was rotated and the apparatus was refit to measure the caudal and lateral orientations.

3 mm of slack was introduced to the cord at the start of each trial to improve the consistency of the initial stick-slip interaction. All friction tests were performed on three surfaces with a predefined roughness. The surfaces were 3D printed grids (PLA, MeltInk, printed on a LulzBot Taz 6) with walls 1 mm thick and 1 mm deep, and different spacing that is normalized by the scale length of a single kirigami skin scale (4.15 mm). The scale length was the same for each skin tested and determined by the row height (*H*). The three surface’s characteristic spacings were 0.5x (2.075 mm), 1x (4.15 mm), and 2x (8.3 mm) scale length (see Figure 4B). As not all skins could enact the same bend angle, each skin was tested in increments of 10° up to its maximum bend angle: 30° for rectilinear with gradient, 50° for curvilinear and curvilinear with the additional circumferential translation, and 70° for polar. This was done to compare the skins against one another, as well as to see how the rotation of scales from bending affected lateral friction.

The caudal:cranial ratio represents the ratio between the caudal (backward) and cranial (forward) drag directions and the lateral-cranial ratio represents the ratio between the lateral and forward drag directions. Figures 4C,D show the lateral-cranial and the caudal-cranial friction ratios, respectively. The friction ratios were calculated using the average frictional force measured during dragging after the static friction peak was overcome. We found curvilinear skins (with and without additional circumferential translation) produced the highest lateral-cranial friction ratios across all surfaces. The additional 2° of scale rotation achieved by circumferential translation resulted in an increase in frictional force compared to the plain curvilinear skins, especially on the larger asperities.

The polar skin, although having scale rotation approaching the ideal vector for lateral pushing did not perform well (friction ratios did not exceed 1.5 on average) on small grid-spacing (asperities were 0.5x the length of the skin scales). Despite the scales having the same length as one another, the width of the scale limited how many scales could engage with asperities. The ventral portion of the polar skin only had one column of scales interacting with the ground whereas the rectilinear and curvilinear skins had three or more columns. We infer that the more scales are in contact with the surface at a time, the higher the friction force.

The curvilinear lattices produced a much higher lateral-cranial friction ratio compared to the rectilinear lattice that had no explicit design intent for increasing lateral resistance. The polar lattice, when on the 2x surface with asperities large enough to engage with, also produced high lateral-cranial ratios at high bend angles. Therefore, the curved lattices, designed to orient the scale tips closer to the ideal vector for lateral pushing, were successful in increasing lateral resistance.

As expected, the rectilinear skin had the highest caudal-cranial friction ratio (exceeding 5:1) as there were more scales pointed longitudinally with respect to the surface asperities. The curved lattices make a trade off to increase lateral friction by decreasing caudal-cranial anisotropy. Despite decreasing the number of scales oriented axially for longitudinal pushing, the curved lattices still marginally exceeded a 1:1 ratio for caudal-cranial anisotropy. Future work could evaluate how the number of anchor points (scales in contact with surface asperities) affects the resulting frictional forces.

### 3.3 Locomotion

**FIGURE 5:**
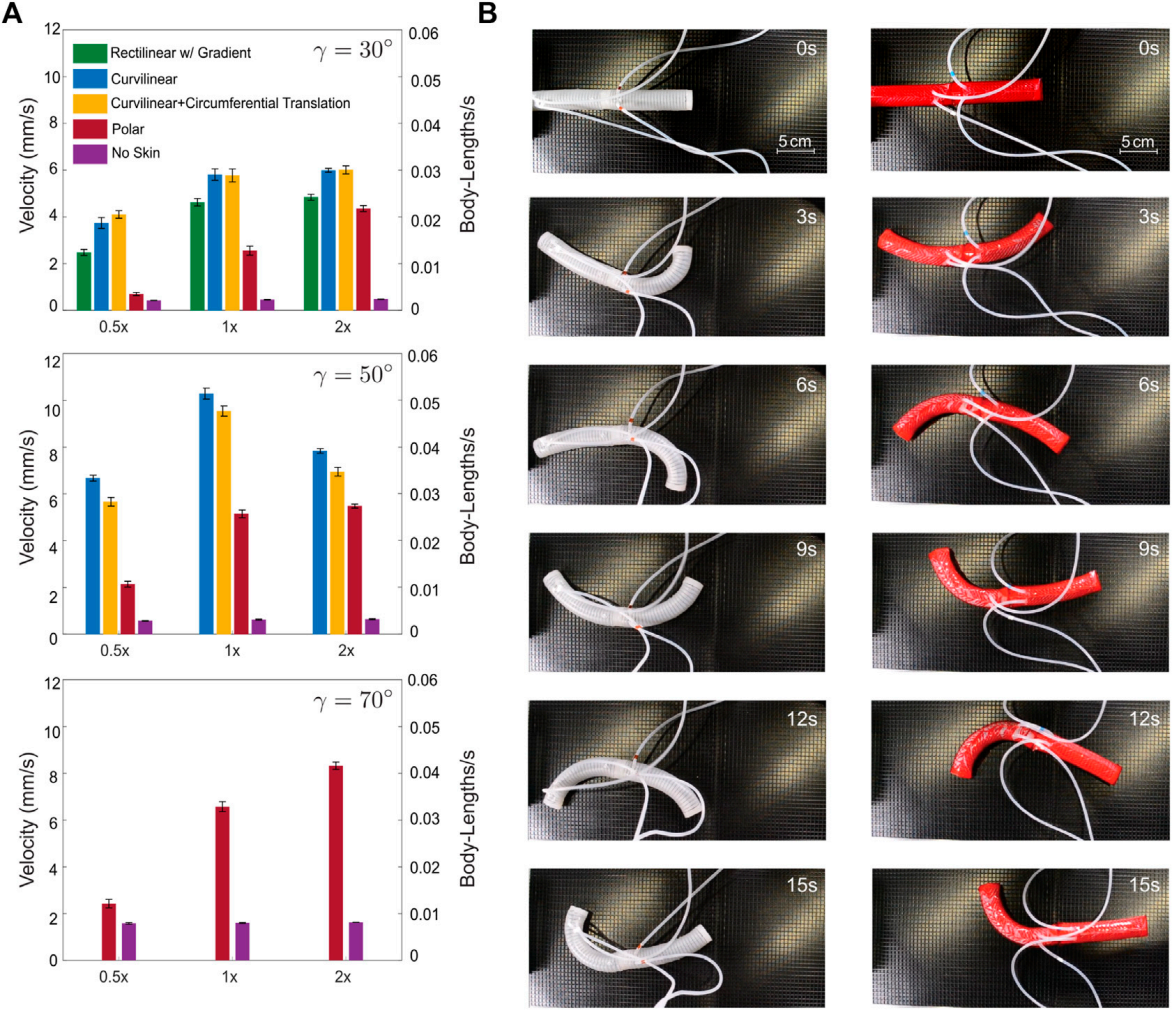
**(A)** Average velocities of skins on varying surfaces. **(B)** Timelapse of soft snake robot without skin and with curvilinear skin on the 1x surface.

Locomotion experiments were performed at each of the maximum bend angles achieved by the different skins and compared to a robot with no skin on all three surfaces. The gait parameters driving lateral undulation are frequency of bending, and amplitude of the bend. The frequency was held constant across all tests. The amplitude was adjusted for each skin to drive the actuators to the desired bend angle determined in Figure 3. Motion capture data was collect by the OptiTrack Prime 13 system (Natural Point Inc.) and used to calculate the velocity of the robot. The average velocity of the robot with each skin is shown in Figure 5A.

Based on the locomotion results, we found that the production of lateral resistance, even to the detriment of the caudal-cranial frictional anisotropy, proved vital in increasing velocity. The curvilinear lattices, which had good rotation of scales and good geometric stiffness of scales (based on our tangible and visual observations) performed best. The polar lattice was not effective on the surfaces with small asperities, so it performed best on the 2x surface, but still had a maximum velocity less than that of the curvilinear skins. Maximizing bend angle maximizes velocity. Despite only bending to 50°, the curvilinear skin increased the velocity of a robot with no skin by 530% at it is maximum velocity which occurred on the 1x surface (time-lapse shown in Figure 5B). Comparing to the rectilinear skin at 30°, both the rectilinear and curvilinear skins did best on the 2x surface with a 25% increase in velocity of the curvilinear skin over the rectilinear skin.

**FIGURE 6:**
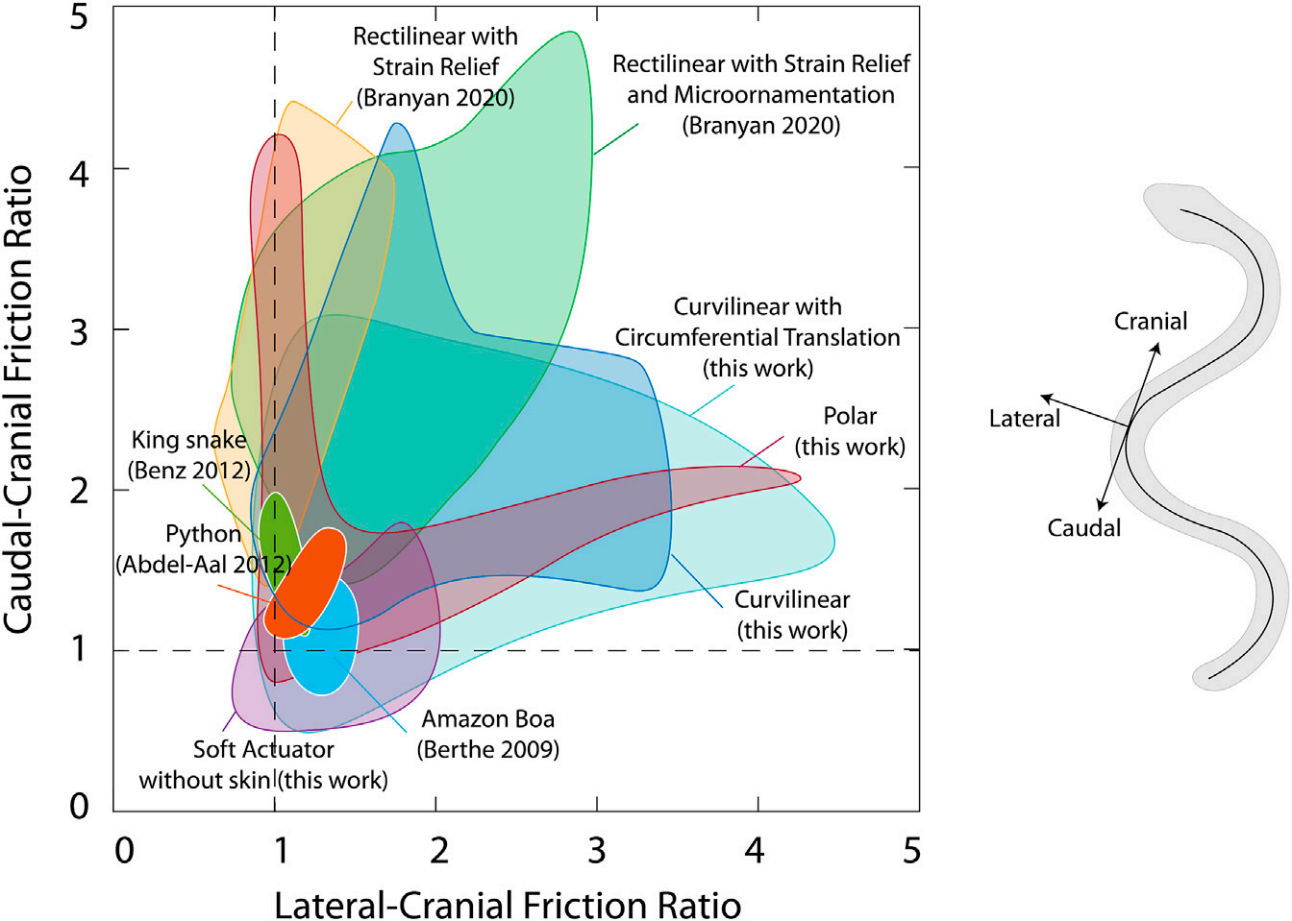
Ashby diagram representing caudal-cranial friction ratio versus lateral-cranial friction ratio for the proposed kirigami skins compared to previous iteration of skin design using microornamentation (Branyan et al., 2020) and biological friction data reported in the literature (Berthe et al., 2009; Benz et al., 2012; Abdel-Aal, 2013)

Figure 6 shows an Ashby plot comparing the frictional ratios of the new curved lattice skins, the skin developed previously in (Branyan et al., 2020), and friction measurements on biological snakes from the literature (Berthe et al., 2009; Benz et al., 2012; Abdel-Aal, 2013). This information can be used to select an appropriate skin for the desired frictional properties. Ideally, any skin on a limbless robot should employ frictional ratios greater than 1. If the selected gait is lateral undulation, where lateral resistance is necessary, targeting larger lateral-cranial ratios is best based on the experiments performed in this work.

## 4 Conclusion

Lateral friction is crucial for lateral undulation in biological snakes Hu et al. (2009). However, in existing snake-inspired robots, other than using wheels, no other mechanism has been proposed to increase lateral resistance over caudal-cranial anisotropy. We introduced here a set of curved kirigami skins that take advantage of the kinematics of bending to orient 3D structures to produce higher lateral reaction forces when engaged with surface asperities. Unlike the typical rectilinear grid pattern, the array of cuts embedded in the curvilinear kirigami skins were arranged along a curve. By using curved rows, the tips of the scales were oriented more laterally, such that bending, of the body would bring the scales further into alignment with the ideal lateral force vector. The curved design also introduced a gradient of hinge widths which reduced the overall axial stiffness of the skin, further facilitating body-bending when wrapped around a soft bending actuator.

We showed when more scales point laterally (minimizing *θ*), a higher lateral reaction force upon contact with an asperity is achieved. The angle of scale rotation coupled with the size and geometric stiffness of the scale drives the locomotion performance. It was found that the curvilinear skins produced the highest speed, improving the velocity over a robot with no skin by 530%. Though the curvilinear skins could not produce as much bending as the polar skin, they accommodated more scales on the ventral portion of the robot to engage with the surface, thus, increasing its overall locomotion efficiency.

In the polar skin, because of the gradient of scale sizes along the longitudinal axis, a non-uniform curvature emerges. This shows how kirigami skins can be used to drive not only the interaction between a robot and its environment, but the kinematics of the robot itself. Work on using kirigami lattice patterns to drive the shape of soft inflatable bladders has been recently introduced (Jin et al., 2020). Future work in using kirigami to drive both the behavior and interaction of soft actuators could greatly reduce the manufacturing effort on the soft actuator side. Kirigami skins can be tailored to achieve the desired kinematics and interaction dynamics necessary to produce the targeted behavior when wrapped around any soft inflatable bladder. Ultimately, we consider this work as contributing to the broader design knowledge of body-skin-environment interactions which will mediate soft robotic performance in potential applications ranging from medical implants to warehouse material handling to planetary surface exploration.

## Data Availability

The original contributions presented in the study are included in the article/Supplementary Material, further inquiries can be directed to the corresponding author.

## References

[B1] Abdel-Aal H. A. (2013). On Surface Structure and Friction Regulation in Reptilian Limbless Locomotion. Journal of the Mechanical Behavior of Biomedical Materials 22, 115–135. 10.1016/j.jmbbm.2012.09.014 23582565

[B2] An N. Domel A. G. Zhou J. Rafsanjani A. Bertoldi K. (2020). Programmable Hierarchical Kirigami. Adv. Funct. Mater. 30, 1906711. 10.1002/ADFM.201906711

[B3] Babaee S. Pajovic S. Rafsanjani A. Shi Y. Bertoldi K. Traverso G. (2020). Bioinspired Kirigami Metasurfaces as Assistive Shoe Grips. Nat Biomed Eng 4, 778–786. 10.1038/s41551-020-0564-3 32483298

[B4] Babaee S. Shi Y. Abbasalizadeh S. Tamang S. Hess K. Collins J. E. (2021). Kirigami-inspired Stents for Sustained Local Delivery of Therapeutics. Nat. Mater. 20, 1085–1092. 10.1038/s41563-021-01031-1 34127823

[B5] Backwater Reptiles Blog (2020). Most Popular Corn Snake Morphs. Rocklin, CA: Backwater Reptiles Blog.

[B6] Benz M. J. Kovalev A. E. Gorb S. N. (2012). “Anisotropic Frictional Properties in Snakes,” in Bioinspiration, Biomimetics, and Bioreplication 2012. Editor Lakhtakia A. (Bellingham, Washington, USA: SPIE), Vol. 8339, 83390X. 10.1117/12.916972

[B7] Berthé R. A. Westhoff G. Bleckmann H. Gorb S. N. (2009). Surface Structure and Frictional Properties of the Skin of the Amazon Tree boa *Corallus hortulanus* (Squamata, Boidae). J Comp Physiol A 195, 311–318. 10.1007/s00359-008-0408-1 PMC2755753 19137315

[B8] Blees M. K. Barnard A. W. Rose P. A. Roberts S. P. McGill K. L. Huang P. Y. (2015). Graphene Kirigami. Nature 524, 204–207. 10.1038/nature14588 26222025

[B9] Branyan C. Fleming C. Remaley J. Kothari A. Tumer K. Hatton R. L. (2017). “Soft Snake Robots: Mechanical Design and Geometric Gait Implementation,” in 2017 IEEE International Conference on Robotics and Biomimetics (ROBIO) (Macau, Macao: IEEE), 282–289. 10.1109/ROBIO.2017.8324431

[B10] Branyan C. Hatton R. L. Menguc Y. (2020). Snake-Inspired Kirigami Skin for Lateral Undulation of a Soft Snake Robot. IEEE Robot. Autom. Lett. 5, 1728–1733. 10.1109/lra.2020.2969949

[B11] Calisti M. Picardi G. Laschi C. (2017). Fundamentals of Soft Robot Locomotion. J. R. Soc. Interface. 14, 20170101. 10.1098/rsif.2017.0101 28539483 PMC5454300

[B12] Crespi A. Jan Ijspeert A. (2006). “AmphiBot II: An Amphibious Snake Robot that Crawls and Swims Using a Central Pattern Generator,” in Proceedings of the 9th International Conference on Climbing and Walking Robots, 19–27.

[B13] Gans C. (1962). Terrestrial Locomotion without Limbs. Am Zool 2, 167–182. 10.1093/icb/2.2.167

[B14] Gray J. Lissmann H. W. (1950). The Kinetics of Locomotion of the Grass-Snake. Journal of Experimental Biology 26, 354–367. 10.1242/jeb.26.4.354

[B15] Hirose S. (1993). Biologically Inspired Robots (Snake-like Locomotor and Manipulator). Oxford: Oxford University Press.

[B16] Hong Y. Chi Y. Wu S. Li Y. Zhu Y. Yin J. (2022). Boundary Curvature Guided Programmable Shape-Morphing Kirigami Sheets. Nat Commun 13. 10.1038/s41467-022-28187-x PMC8792031 35082311

[B17] Hu D. L. Nirody J. Scott T. Shelley M. J. (2009). The Mechanics of Slithering Locomotion. Proc. Natl. Acad. Sci. U.S.A. 106, 10081–10085. 10.1073/pnas.0812533106 19506255 PMC2700932

[B18] Isobe M. Okumura K. (2016). Initial Rigid Response and Softening Transition of Highly Stretchable Kirigami Sheet Materials. Sci Rep 6, 24758. 10.1038/srep24758 27117355 PMC4846813

[B19] Jin L. Forte A. E. Deng B. Rafsanjani A. Bertoldi K. (2020). Kirigami‐Inspired Inflatables with Programmable Shapes. Adv. Mater. 32, 2001863. 10.1002/adma.202001863 32627259

[B20] Lamoureux A. Lee K. Shlian M. Forrest S. R. Shtein M. (2015). Dynamic Kirigami Structures for Integrated Solar Tracking. Nat Commun 6, 8092. 10.1038/ncomms9092 26348820 PMC4569711

[B21] Liu B. Ozkan-Aydin Y. Goldman D. I. Hammond F. L. III (2019). “Kirigami Skin Improves Soft Earthworm Robot Anchoring and Locomotion under Cohesive Soil,” in IEEE International Conference on Soft Robotics, 828–833. 10.1109/robosoft.2019.8722821

[B22] Marvi H. Cook J. P. Streator J. L. Hu D. L. (2016). Snakes Move Their Scales to Increase Friction. Biotribology 5, 52–60. 10.1016/J.BIOTRI.2015.11.001

[B23] Marvi H. Hu D. L. (2012). Friction Enhancement in Concertina Locomotion of Snakes. J. R. Soc. Interface. 9, 3067–3080. 10.1098/rsif.2012.0132 22728386 PMC3479897

[B24] Onal C. D. Rus D. (2013). Autonomous Undulatory Serpentine Locomotion Utilizing Body Dynamics of a Fluidic Soft Robot. Bioinspir. Biomim. 8, 026003–026010. 10.1088/1748-3182/8/2/026003 23524383

[B25] Rafsanjani A. Bertoldi K. Studart A. R. (2019a). Programming Soft Robots with Flexible Mechanical Metamaterials. Sci Robot 4, 7874. 10.1126/scirobotics.aav7874 33137714

[B26] Rafsanjani A. Bertoldi K. Paulson J. A. (2017). Buckling-Induced Kirigami. Phys. Rev. Lett. 118. 10.1103/PhysRevLett.118.084301 28282190

[B27] Rafsanjani A. Jin L. Deng B. Bertoldi K. (2019b). Propagation of Pop Ups in Kirigami Shells. Proc. Natl. Acad. Sci. U.S.A. 116, 8200–8205. 10.1073/pnas.1817763116 30962388 PMC6486746

[B28] Rafsanjani A. Zhang Y. Liu B. Rubinstein S. M. Bertoldi K. (2018). Kirigami Skins Make a Simple Soft Actuator Crawl. Sci. Robot. 3, eaar7555. 10.1126/scirobotics.aar7555 33141681

[B29] Shyu T. C. Damasceno P. F. Dodd P. M. Lamoureux A. Xu L. Shlian M. (2015). A Kirigami Approach to Engineering Elasticity in Nanocomposites through Patterned Defects. Nature Mater 14, 785–789. 10.1038/nmat4327 26099109

[B30] Yang Y. Vella K. Holmes D. P. (2021). Grasping with Kirigami Shells. Science Robotics 6, abd6426. 10.1126/scirobotics.abd6426 34043535

[B31] Zhang X. Naughton N. Parthasarathy T. Gazzola M. (2021). Friction Modulation in Limbless, Three-Dimensional Gaits and Heterogeneous Terrains. Nat Commun 12, 1–8. 10.1038/s41467-021-26276-x 34667170 PMC8526626

[B32] Zhang Y. Yan Z. Nan K. Xiao D. Liu Y. Luan H. (2015). A Mechanically Driven Form of Kirigami as a Route to 3D Mesostructures in Micro/nanomembranes. Proc. Natl. Acad. Sci. U.S.A. 112, 11757–11764. 10.1073/pnas.1515602112 26372959 PMC4586832

