# Supplementary material for: Curvilinear Kirigami Skins Let Soft Bending Actuators Slither Faster
Source: Front Robot AI. 2022 May 3;9:872007. doi: 10.3389/frobt.2022.872007 (PMC9110809; doi:10.3389/frobt.2022.872007)
Supplement: Supplementary file 1 [file DataSheet1.pdf]

Supplementary Material

1 LATTICE GEOMETRY

The definitions of the three lattices are shown in Fig. S1a. Algorithms used to generate the hinge locations for each lattice can be found below. The rectilinear lattice refers to the typical kirigami lattice structure of a parallel grid aligning the hinges and profiles with the direction of the applied axial strain. As this is the standard kirigami pattern, there is no strategy for increasing lateral friction other than the rotation of the scales with respect to surface asperities inherent upon the bending of the actuator. This lattice is defined by a lattice length, L, lattice angle, ϕ, and a hinge width, δ. The geometric relationship between these parameters result in a uniform column width, $W = l\cos(\phi/2)$ and a row height of scales $H = l\sin(\phi/2)$. The hinge locations can be identified by (x, y) coordinates based on the values of the described parameters. Since we are comparing this standard kirigami lattice to our newly created lattices, maintaining consistent parameter values is necessary. The following lattices are built based on these described parameter values.

The polar lattice is defined by polar coordinates rather than rectilinear coordinates. A radius R defines the shape of the rows which are offset by the same row height H defined by the rectilinear lattice. An angle, $\theta_{lattice}$ defines the orientation and spacing of the columns. The hinge locations are defined by the $(R, \theta_{lattice})$ coordinates. An additional parameter, K, defines a negative y-displacement from the origin (center, bottom of the lattice pattern shown in Fig S1a). of the polar lattice in which to define the center point of the radius. This is necessary because as you move axially along the lattice, the columns get closer together. This parameter K controls the smallest and largest scale size along the length of the lattice. The lower bound of K is limited by the point at which scales will intersect one another, and the lattice structure would breakdown. The change in scale size along the skin complicates our implementation of a soft snake robot as this creates a gradient of stiffness along the longitudinal axis which results in non-constant curvature. More importantly, the larger the scale, the less stiff it is, so interactions with an asperity on a surface will be relatively weak as the scale size increases. The specific micro-mechanics of asperity-scale interaction is beyond the scope of the current work, but opens up intriguing new mechanical design questions, as detailed in the discussion and conclusion.

The curvilinear lattice is a combination of the rectilinear and polar lattices. To prevent the change in scale size along the skin as is apparent with the polar lattice, the parallel columns from the rectilinear lattice were maintained while curved rows from the polar lattice were implemented. The rows of scales are defined by a radius, R and the columns are defined by the same column width W from the rectilinear lattice. Hinge locations are defined by the intersection of the radius, with the parallel columns.

As the goal is to maximize lateral friction by orienting the scales as close to lateral as possible, an additional circumferential translation of the scale tips was implemented on the curvilinear lattice and is defined by arc angle $\zeta = 5°$ as shown in Fig. S1b. As the ventral and part of the ventro-lateral portions of the skin are the only parts in contact with surface asperities, that was the only area of the curvilinear lattice where additional scale tip circumferential translations were implemented. Performing a circumferential translation of the scales caused a decrease in hinge width that would lead to the intersection of cuts if

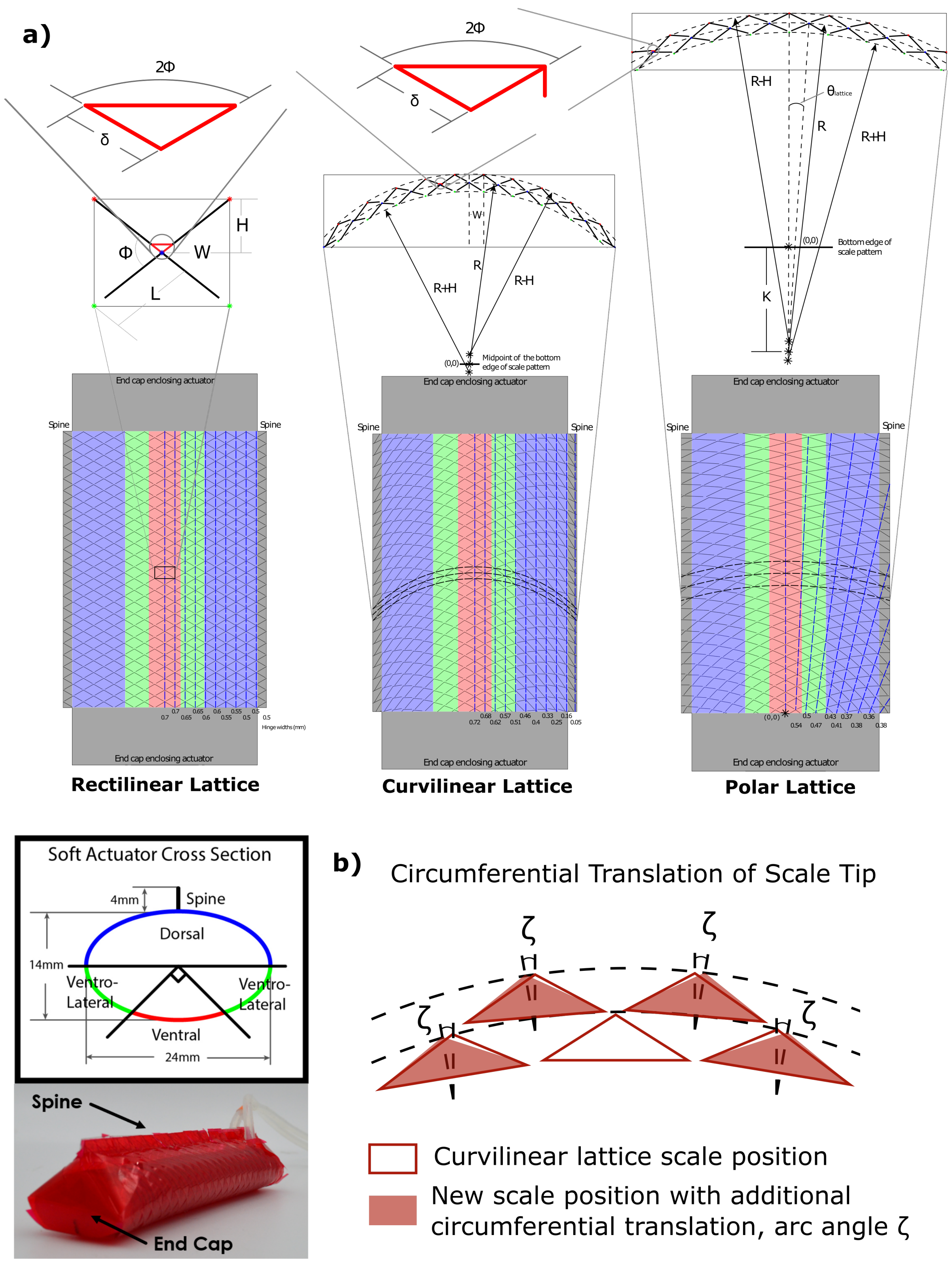

Figure S1: **a)** Kirigami lattice patterns with broken out unit cell and hinge definitions. **b)** Additional circumferential translation parameter used on curvilinear lattice to further push scale tip towards lateral ideal.

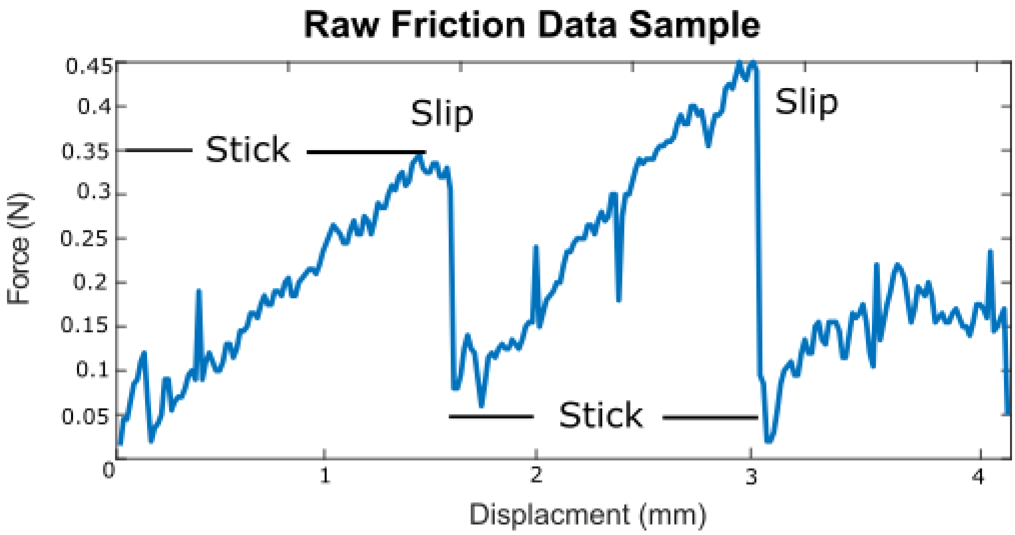

Figure S2: Plot of raw data collected from drag experiments to determine friction of skins on actuator.

propagated across the entire width of the skin. By keeping the additional circumferential translation of the scales to high interaction locations, the integrity of the skin is maintained.

2 FRICTION DATA PROCESSING

The drag experiments described in the manuscript resulted in force vs. displacement data. This experimental data was used to calculate friction in the cranial, caudal, and lateral directions of the skin as it was pulled across a rough surface. The scales on the skin would engage with an asperity on the surface producing high reaction forces until a maximum was reached and the scale disengaged from the asperity. This results in a stick-slip phenomenon. The plot in Fig. S2 shows a sample of the force-displacement data collected illustrating stick-slip. We took the average over several iterations of stick-slip as a way to estimate dynamic friction. We then took the average over ten trials for each skin, bend angle, and surface combination. The caudal friction data was divided by the cranial friction data for each combination to produce the caudal:cranial friction ratio. The same was done for the lateral friction data to produce the lateral:cranial ratio.

3 FRICTION RATIO WITH ROBOT WITHOUT SKIN

The plots shown in Fig. S3 provide a comparison of the frictional ratios of the skins designed in this work with a robot without skin. It is difficult to make a direct comparison because the surface interaction of the robot without skin is adhesion, which relies on surface area engagement. As seen in the plots, there is no frictional anisotropy (ratios greater than 1) present in the robot without skin for the caudal-cranial ratio. The lateral-cranial ratio shows some frictional anisotropy for the lower bend angles because more of the surface was oriented perpendicular to the drag direction. Due to the effects of adhesion, this increased the drag force of the robot as it was pulled across the test surface. Biological snakes rely on sliding friction as their primary surface interaction which is what motivated this work. The skins were designed to remove any effects from adhesion, and introduce frictional anisotropy present on biological snake skin.

4 ALGORITHMS

The algorithms shown below outline the method for generating the hinge locations for both the unit cell, and the full lattice, of each lattice type. The values of R and $\theta_{lattice}$ are used to determine which lattice

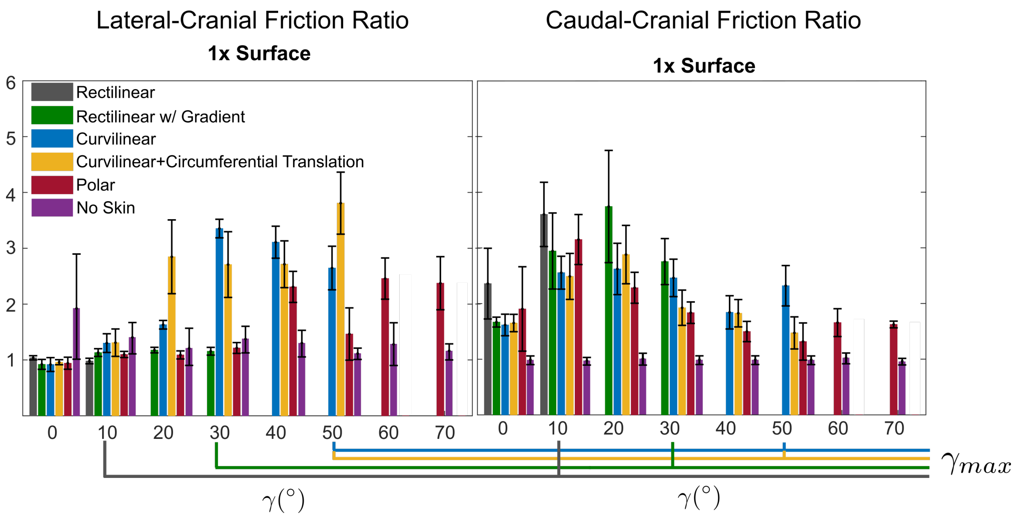

Figure S3: Plots of anisotropic friction ratios including robot without skin

is being used. If $R \to \infty$ that implies that the rows are horizontal lines rather than radii, and therefore reference the typical rectilinear lattice. Any other value of R can be used to generate one of the curved lattices. $\theta_{lattice}$ is then used to determine whether the desired lattice is curvilinear or polar. If $\theta_{lattice} = 0$ then that means the columns are vertical as they are in the rectilinear lattice, which then determines that the desired lattice is curvilinear. Any other value of $\theta_{lattice}$ can be used to generate the polar lattice.

4.1 Notes for Algorithms 2 and 3

Nested FOR loops, as used for the polar lattice algorithm, can be used for patterning the unit cells for the other two lattices. We left them broken out here to emphasize how the unit cell is defined. Using the nested FOR loops simplifies the definition of the Middle_Hinges[1](X,Y) as the first (X,Y) pair in the Middle_Hinges array is the center point of the unit cell that then gets translated to make the rest of the tessellated structure. However, leaving the unit cell as a separate definition helps to show that only the curvilinear and rectilinear lattices have unit cells whereas the polar lattice does not.

4.2 Notes for Algorithms 3 and 4

The curvilinear lattice is a hybrid of the rectilinear lattice and the polar lattice. That means that the linear columns from the rectilinear lattice are maintained with curvilinear rows. Because we started with the rectilinear lattice, we carry over the column and row spacing as defined there ($X_{off} = 2l\cos(\phi/2)$ and $Y_{off} = l\sin(\phi/2)$) to compare them directly in use on the robot. However, this is arbitrary. Both the column and row spacing can be chosen at will. The rectilinear lattice is defined by a lattice width l and lattice angle ϕ, which then define the column and row spacing. The selection of l and ϕ is arbitrary though. There is no reason the column and row spacing have to be the same across lattices, that's just what we did to make a proper comparison in performance. For those not starting from the rectilinear lattice, using l and ϕ doesn't make sense. Instead, X_{off} and Y_{off} values can be defined directly. This is also true for the polar lattice which departs even further from the original rectilinear lattice strategy.

Algorithm 1: Hinge Locations for all Lattices

Input: $l, \phi, R, \theta_{lattice}, M, N, K$
Output: (X,Y) Coordinates for the hinge locations for selected lattice

Parameters
l = lattice spacing ϕ = lattice angle
R = radius $\theta_{lattice}$ = angle between columns
M = skin width N = skin length

K = negative y-displacement from origin for center of radii

$X_{off} = 2l\cos(\phi/2)$: distance between columns

$Y_{off} = l\sin(\phi/2)$: distance between rows

(X,Y) Output Arrays
Top_Hinges [ ] : array of hinge locations for middle row
Middle_Hinges [ ] : array of hinge locations for middle row
Bottom_Hinges [ ] : array of hinge locations for middle row

if $\theta_{lattice} = 0$ && $R \to \infty$ **then**
| rectilinear_lattice(X_{off}, Y_{off}, M, N)
end

if $\theta_{lattice} = 0$ **then**
| curv_lattice($R, X_{off}, Y_{off}, M, N$)
end

else
| pol_lattice($R, \theta_{lattice}, Y_{off}, M, N, K$)
end

Algorithm 2: rectilinear_lattice (X_{off}, Y_{off}, M, N)

Result: (X,Y) Coordinates for the hinge locations on the rectilinear lattice

Unit Cell Definition

Middle Hinge [1] = (0 , 0)

Top_Hinges [1] = $(X_{off}/2, Y_{off})$;

Top_Hinges [2] = $(-1 * X_{off}/2, Y_{off})$;

Bottom_Hinges [1] = $(X_{off}/2, -1 * Y_{off})$;

Bottom_Hinges [2] = $(-1 * X_{off}/2, -1 * Y_{off})$;

Pattern unit cells along width and length of skin

for *m = 1:M (rounded to nearest integer)* **do**
| **for** *n = 1:N (rounded to nearest integer* **do**
| | (Middle_Hinges [1]_X += $X_{off} * m$, Middle_Hinges [1]_Y += $2Y_{off} * n$) ;
| **end**
end

Algorithm 3: curv_lattice $(R, X_{off}, Y_{off}, M, N)$

Result: (X,Y) Coordinates for the hinge locations on the curvilinear lattice

Unit Cell Definition

Middle_Hinges [1] = (0 , r)

for *i = 1:1/2M /* X_{off} *(rounded to nearest integer)* **do**

  Middle_Hinges_Right [i+1] = $(X_{off} * i, \sqrt{R^2 - (X_{off} * i)^2})$;

  Middle_Hinges_Left [i+1] = $(-X_{off} * i, \sqrt{R^2 - (X_{off} * i)^2})$;

  Top_Hinges_Right [i+1] = $(X_{off} * i - X_{off}/2, \sqrt{R^2 - (X_{off} * i - X_{off}/2)^2} + Y_{off})$;

  Top_Hinges_Left [i+1] = $(-X_{off} * i - X_{off}/2, \sqrt{R^2 - (X_{off} * i - X_{off}/2)^2} + Y_{off}$;

  Bottom_Hinges_Right [i+1] = $(X_{off} * i - X_{off}/2, \sqrt{R^2 - (X_{off} * i - X_{off}/2)^2} - Y_{off})$;

  Bottom_Hinges_Left [i+1] = $(-X_{off} * i - X_{off}/2, \sqrt{R^2 - (X_{off} * i - X_{off}/2)^2} - Y_{off})$;

end

Middle_Hinges = [Middle_Hinges_Right Middle_Hinges_Left];
Top_Hinges = [Top_Hinges_Right Top_Hinges_Left];
Bottom_Hinges = [Bottom_Hinges_Right Bottom_Hinges_Left];

Pattern unit cells along length of skin

for *i = 1:N (rounded to nearest integer)* **do**

  (Middle_Hinges [1]_X, Middle_Hinges [1]_Y += $2 * Y_{off}$);

end

Algorithm 4: pol_lattice $(R, \theta_{lattice}, Y_{off}, M, N, K)$

Result: (X,Y) Coordinates for the hinge locations on the polar lattice

Full lattice pattern generation (unit cell undefinable)

for *n = 0:N (round to nearest integer)* **do**

  $R = R + Y_{off} * n$;

  for *m = 0:1/2M /* $R \sin \theta_{lattice}$ *(rounded to nearest integer)* **do**

    i = odd numbered m's;

    Middle_Hinges_Right [m] =
    $((R - Y_{off}) \sin(\theta_{lattice}/2 * i), (R - Y_{off}) \cos(\theta_{lattice}/2 * i) - K)$;

    Middle_Hinges_Left [m] =
    $((R - Y_{off}) \sin(-\theta_{lattice}/2 * i), (R - Y_{off}) \cos(\theta_{lattice}/2 * i) - K)$;

    Top_Hinges_Right [m+1] = $(R \sin(\theta_{lattice} * m), R \cos(\theta_{lattice} * m) - K)$;

    Top_Hinges_Left [m+1] = $(R \sin(-\theta_{lattice} * m), R \cos(\theta_{lattice} * m) - K)$;

    Bottom_Hinges_Right [m+1] =
    $((R - Y_{off} * 2) \sin(\theta_{lattice} * m), (R - Y_{off} * 2) \cos(\theta_{lattice} * m) - K)$;

    Bottom_Hinges_Left [m+1] =
    $((R - Y_{off} * 2) \sin(-\theta_{lattice} * m), (R - Y_{off} * 2) \cos(\theta_{lattice} * m) - K)$;

  end

end

Middle_Hinges = [Middle_Hinges_Right Middle_Hinges_Left];
Top_Hinges = [Top_Hinges_Right Top_Hinges_Left];
Bottom_Hinges = [Bottom_Hinges_Right Bottom_Hinges_Left];
